# Idiotope-Driven T-Cell/B-Cell Collaboration-Based T-Cell Epitope Prediction Using B-Cell Receptor Repertoire Sequences in Infectious Diseases

**DOI:** 10.3390/v15051186

**Published:** 2023-05-17

**Authors:** Yukio Nakamura, Meng Ling Moi, Takashi Shiina, Tadasu Shin-I, Ryuji Suzuki

**Affiliations:** 1 Repertoire Genesis Inc., Osaka 567-0085, Japan; 2 Department of Developmental Medical Sciences, Graduate School of Medicine, The University of Tokyo, Tokyo 113-0033, Japan; 3 Department of Molecular Life Science, Tokai University School of Medicine, Kanagawa 259-1193, Japan; 4 BITS Co., Ltd., Tokyo 101-0062, Japan; 5 Department of Rheumatology and Clinical Immunology, Clinical Research Center for Rheumatology and Allergy, National Hospital Organization Sagamihara National Hospital, Kanagawa 252-0392, Japan

**Keywords:** T-cell epitope, antigen, idiotype network, repertoire analysis, anti-idiotypic antibody, idiotope-driven T-B collaboration, molecular mimicry, B-cell

## Abstract

T-cell recognition of antigen epitopes is a crucial step for the induction of adaptive immune responses, and the identification of such T-cell epitopes is, therefore, important for understanding diverse immune responses and controlling T-cell immunity. A number of bioinformatic tools exist that predict T-cell epitopes; however, many of these methods highly rely on evaluating conventional peptide presentation by major histocompatibility complex (MHC) molecules, but they ignore epitope sequences recognized by T-cell receptor (TCR). Immunogenic determinant idiotopes are present on the variable regions of immunoglobulin molecules expressed on and secreted by B-cells. In idiotope-driven T-cell/B-cell collaboration, B-cells present the idiotopes on MHC molecules for recognition by idiotope-specific T-cells. According to the idiotype network theory formulated by Niels Jerne, such idiotopes found on anti-idiotypic antibodies exhibit molecular mimicry of antigens. Here, by combining these concepts and defining the patterns of TCR-recognized epitope motifs (TREMs), we developed a T-cell epitope prediction method that identifies T-cell epitopes derived from antigen proteins by analyzing B-cell receptor (BCR) sequences. This method allowed us to identify T-cell epitopes that contain the same TREM patterns between BCR and viral antigen sequences in two different infectious diseases caused by dengue virus and SARS-CoV-2 infection. The identified epitopes were among the T-cell epitopes detected in previous studies, and T-cell stimulatory immunogenicity was confirmed. Thus, our data support this method as a powerful tool for the discovery of T-cell epitopes from BCR sequences.

## 1. Introduction

T-cell epitope recognition is fundamental to inducing proper and robust cellular and humoral immune responses during adaptive immunity induced by various triggers, including infectious disease, allergy, autoimmunity, transplantation, and cancer [1]. Short peptides recognized by T-cells, termed T-cell epitopes, are presented as a complex associated with the groove of major histocompatibility complex (MHC) molecules expressed on the surface of antigen-presenting cells (APCs). Complexes of the T-cell epitope and MHC elicit distinct T-cell responses depending on different MHC classes [2,3]. MHC class I usually binds short peptides (e.g., 9-mer) and activates CD8^+^ T-cells to differentiate into cytotoxic T-cells (CTLs) during cellular immunity. By contrast, MHC class II interacts with slightly longer peptides (e.g., 15-mer), which are mainly expressed on professional APCs, including dendritic cells, macrophages, and B-cells. These cells take up exogenous antigens to stimulate the differentiation of CD4^+^ T-cells into helper T-cells. CD4^+^ helper T-cells producing interferon (IFN)-γ and interleukin (IL)-2 promote CTL responses, whereas helper T-cells producing IL-4, IL-5, and IL-13 promote the activation and differentiation of B-cells, resulting in antibody production in humoral immune responses.

The identification of T-cell epitopes enables a mechanistic understanding of various diseases and immune responses, which might help improve vaccine designs. Given the vast diversity of antigens, polymorphisms in MHC and the T-cell populations of individuals, bioinformatic analysis is a powerful tool for predicting T-cell epitopes. Many in silico methods of T-cell epitope prediction have been developed [1,4]. However, many of these are highly dependent on evaluating the probability of possible target epitope sequences for MHC binding and antigen processing, and do not focus on more direct epitope sequences scanned by T-cell receptors (TCRs).

**Figure 1:**
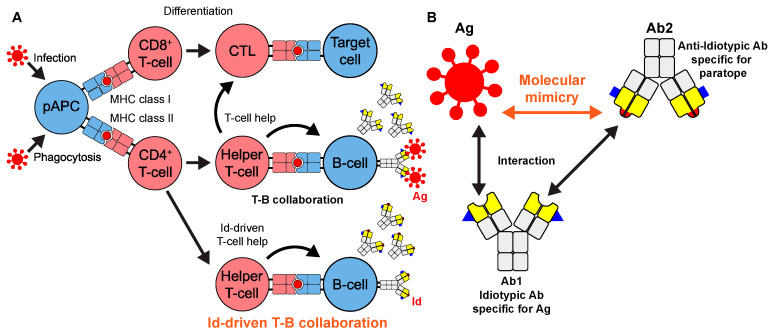
Two forms of T-B collaboration and molecular mimicry. (**A**) Infecting antigens or antigens taken up by phagocytosis in a professional antigen-presenting cell (pAPC) are processed and presented as peptides (red) on the MHC molecule to be recognized by specific T-cells. The activated T-cells recognize a peptide (T-cell epitope) on a target cell or B-cell and exert effector functions as cytotoxic T lymphocytes (CTLs) in cellular immunity and as helper T-cells both in cellular and humoral immunity, respectively. In T-B collaboration, the helper T-cells recognize an antigen-derived peptide (red) and promote the production of antibodies against the antigen, while in idiotope (id)-driven T-B collaboration, they recognize an idiotypic peptide (red) and help B-cells produce antibodies containing ids (red). (**B**) Molecular mimicry. Antibody 1 idiotypic antibody (Ab1) is specific for an antigen (Ag). Anti-idiotypic antibody Ab2 will be induced as a consequence of idiotypic interactions and will recognize Ab1. Ab2 is specific for the paratope of Ab1, and thus, exhibits molecular mimicry with the Ag. Analysis of Ab2 sequences will thus identify Ag-derived sequences.

To establish a new T-cell epitope prediction strategy and take advantage of data generated by TCR/B-cell receptor (BCR) repertoire sequencing, we focused on: (1) Idiotope-driven T-cell/B-cell (T-B) collaboration; (2) The immune network theory proposed by Jerne [5] (Figure 1).

First, the so-called T-B collaboration is the interaction of a T-cell and B-cell during antibody production, where the B-cell captures antigens on the BCR expressed on the plasma membrane, degrades them, and presents the resulting peptide on MHC class II to cognate CD4^+^ T-cells, which recognize and are already activated by the same antigen, and pass activation signals to the B-cells (Figure 1A) [6]. In contrast, idiotope-driven T-B collaboration is characterized as a specialized T-B collaboration, involving idiotopes, immunogenic determinants produced in the variable regions of immunoglobulin (Ig) molecules [7]. B-cells in idiotope-driven T-B collaboration display peptides derived from their own Ig proteins to idiotope-specific T-cells, resulting in the production of idiotypic antibodies, rather than antibodies against antigens.

Second, according to Jerne’s immune network theory [5], B-cells form a network of Igs through idiotypic interactions (Figure 1B). Each antigen-specific BCR sequence is created by random V(D)J recombination and somatic hypermutation (SHM) [8]. The resulting BCRs display a novel sequence comprising one specific set of idiotopes, the idiotype, which determines the specificity of the antigen binding sites (paratope). Because of the complementary structures to antigens, the idiotopes of a given antibody (Ab1) can become immunogenic and stimulate the production of new antibodies with complementary idiotypes of Ab1 (Ab2, anti-idiotype). These idiotypic interactions lead to sequential reactions and the formation of antibody networks. Indeed, anti-idiotypic antibodies exist in organisms that recognize idiotopes of different antibodies [9,10,11,12,13]. An anti-idiotypic antibody specific for and binding to the paratope of an idiotypic antibody exhibits the molecular mimicry of the paratope to an antigen.

Evidence for idiotope-driven T-B collaboration was reported in human diseases and mouse models [14,15,16,17,18,19,20,21,22,23,24,25]. Given the presence of anti-idiotypic antibodies, we hypothesized that the analysis of BCR (anti-idiotypic antibody) sequences to determine the molecular mimicry of idiotopes and binding of idiotopes to MHC will identify T-cell epitopes that activate T-cells that recognize idiotopes, and thus, the original antigen.

Here, we defined TCR-recognized epitope motif patterns and developed a new T-cell epitope prediction pipeline that performed a comprehensive search for full-length (FL) BCR sequences containing the epitope motif patterns that were present in the reference antigen sequences. This method identified the functional T-cell epitopes present during infectious diseases, including dengue fever and severe acute respiratory syndrome coronavirus 2 (SARS-CoV-2) infection. This prediction method will be applicable to other diseases using different reference sequences, and thus, will be important for the identification of T-cell epitopes present during various immune responses.

## 2. Materials and Methods

### 2.1. Sample Collection

Peripheral blood mononuclear cells (PBMCs) were collected from patients with dengue virus (DENV) and SARS-CoV-2 infections [26,27,28].

### 2.2. HLA Typing

The HLA alleles of patients were typed for loci of classes I and II, as described previously [29,30].

### 2.3. Peptide Synthesis

Peptides used in the ELISPOT assay were synthesized by GenScript Japan (Tokyo, Japan) with a purity of ≥95%.

### 2.4. PBMCs

Human PBMCs, isolated from two healthy donors, were purchased from Lonza (Basel, Switzerland) and used for ELISPOT assays.

### 2.5. ELISPOT Assays

Cryopreserved PBMCs were thawed as previously reported [31]. Cultured ELISPOT assays were performed as follows. Cells were plated in each well of a 24-well flat-bottom tissue culture plate (5.0 × 10^6^ per mL) and cultured at 37 °C in a 5% CO_2_ atmosphere for 14 days in RPMI 1640 media containing L-Gln and HEPES (Nacalai Tesque, Kyoto, Japan), 10% heat-inactivated fetal bovine serum (FBS), 1 × penicillin-streptomycin-amphotericin B suspension, 10 U/mL human IL-2 (Proteintech), and 1 µg/mL peptide. On Days 3, 7, 10, and 12, half of the culture medium was replaced with fresh RPMI 1640 supplemented with 10% FBS, 1 × penicillin-streptomycin-amphotericin B suspension, 20 U/mL human IL-2, and 2 µg/mL peptide. On Day 15, cells were washed three times with RPMI 1640 without serum. To detect cytokine production in cultured and ex vivo ELISPOT assays, cells were resuspended in 1 mL RPMI 1640 without serum; then, 100 µL of the cell suspension (~4 × 10^5^ cells) was plated in each well of ELISPOT plates containing 100 µL of RPMI 1640 without serum supplemented with or without 20 µg/mL peptide (final 10 µg/mL) or 4 µg/mL (final 2 µg/mL) phytohemagglutinin (Sigma). ELISPOT assays were conducted with human IFN-γ/IL-4 double-color ELISPOT (CTL) in accordance with the manufacturer’s instructions. The membranes were punched out with an acryl device ELI8 [32] and scanned at a resolution of 4800 dpi with a flat head scanner LiDE400 (Canon). Spots were counted from digitalized images using Fiji software [33], adjusted to the number per 1 × 10^5^ cells, and the mean of duplicates was calculated. The numbers of IFN-γ or IL-4 spots were calculated by subtracting the mean numbers of unstimulated cell spots from the mean number of peptide-stimulating cell spots. Cultured ELISPOT assays resulted in high background responses of IFN-γ, probably because of IL-2 in culture medium, and thus, responses were considered positive if the number of spots was at least 10 after subtracting the no-peptide background (most background values were 0–10 spots per well).

### 2.6. Sequence and Epitope Data Collection

Human Ig sequences (version 3.1.29) were obtained from the international ImMunoGeneTics information system (IMGT) (https://www.imgt.org/, accessed on 10 May 2023) [34]. Human protein-coding transcript translation sequences (GENCODE release 36) were downloaded from GENCODE (https://www.gencodegenes.org/, accessed on 10 May 2023) [35], and TCR and Ig genes were excluded to create the human proteome dataset. Genome sequences of DENV and SARS-CoV-2 isolates from infected patients in Vietnam were previously determined [27,28]. For other DENV isolates collected in Vietnam, including 880 DENV-1, 179 DENV-2, 49 DENV-3, and DENV-4, and other flaviviruses, including ZIKA virus, Japanese encephalitis virus (JEV), West Nile virus (WNV), yellow fever virus (YFV), tick-borne encephalitis virus (TBEV), Aedes flavivirus (AEDES), bovine viral diarrhea virus 1 (BVDV), and hepatitis C virus (HCV), the sequences were downloaded from the Virus Variation Resource (https://www.ncbi.nlm.nih.gov/genome/viruses/variation, accessed on 10 May 2023) [36]. Other sequences of SARS-CoV-2 variants and related coronaviruses were obtained from GenBank and GISAID (https://www.gisaid.org/, accessed on 10 May 2023) [37]. DENV and SARS-CoV-2 epitope datasets were obtained from the Immune Epitope Database (IEDB) (http://www.iedb.org, accessed on 10 May 2023) [38].

### 2.7. TCR–Peptide–MHC Structure Analysis

Structures of MHC–peptide–TCR complexes were downloaded in June, 2021, from the PDBj database (https://pdbj.org/, accessed on 10 May 2023) [39]. Supplementary Data File S1 presents the details of these structures and the results of analyses. The number of atomic contacts in a peptide–TCR complex was analyzed using CONTACT version 7.1.014 (https://www.ccp4.ac.uk/html/contact.html, accessed on 10 May 2023). From the resulting data, a distance within 4.5 Å, including a hydrogen bond (~3.5 Å), a van der Waals force (~4.0 Å), and a salt bridge (~4.5 Å), was selected, and the number of atomic contacts per peptide position was determined for each structure. Then, the number of contacts for each position and the frequency of atomic contacts per position were calculated. Positions with a frequency of ≥10% were considered as forming a TCR-recognized epitope motif.

### 2.8. Repertoire Analysis

BCR repertoire analysis was performed as described previously [40]. Briefly, total RNA isolated from PBMCs were used to generate cDNA and perform adaptor-ligation PCR using adaptor-specific primers and BCR (IgM and IgG) C-region-specific primers. Index sequences were added to the PCR products using Nextera XT index kit v2, and the sequence was performed with the Illumina Miseq (2 × 300 bp). The subsequent data processing for characterizing clones was performed using the software Repertoire Genesis developed by Repertoire Genesis Inc.

### 2.9. MHC Binding Prediction

The MHC binding of 9-mer and 15-mer peptide sequences was predicted using NetMHCpan-4.0 [41] for MHC class I and NetMHCIIpan-3.2 [42] for MHC class II, respectively. These tools are trained on binding affinity data, and predict half maximal inhibitory concentration (IC_50_) values (nM) for peptides binding to specific MHC molecules. As a guideline at IEDB (https://www.iedb.org, accessed on 10 May 2023), peptides with IC_50_ values < 50 nM are considered high affinity, <500 nM, intermediate affinity, and <5000 nM, low affinity. The IC_50_ values for each peptide were calculated against all available MHC alleles, including 886 HLA-A, 1412 HLA-B, and 617 HLA-C alleles for NetMHCpan-4.0, and 660 HLA-DRB, 2048 HLA-DP, and 2912 HLA-DQ alleles for NetMHCIIpan-3.2.

### 2.10. B-Cell Epitope Prediction

B-cell epitope prediction was performed using BepiPred-2.0 with the default threshold value (0.5) and FL BCR sequences as input protein sequences. BepiPred-2.0 is based on a random forest algorithm trained on epitopes annotated from antibody–antigen protein structures and predicts B-cell epitopes from a protein sequence [43]. If the score was >0.5, the amino acids were regarded as having B-cell linear epitope probability.

## 3. Results

### 3.1. Determination of T-Cell Epitope Motif Patterns from Peptide–TCR Structures

**Figure 2:**
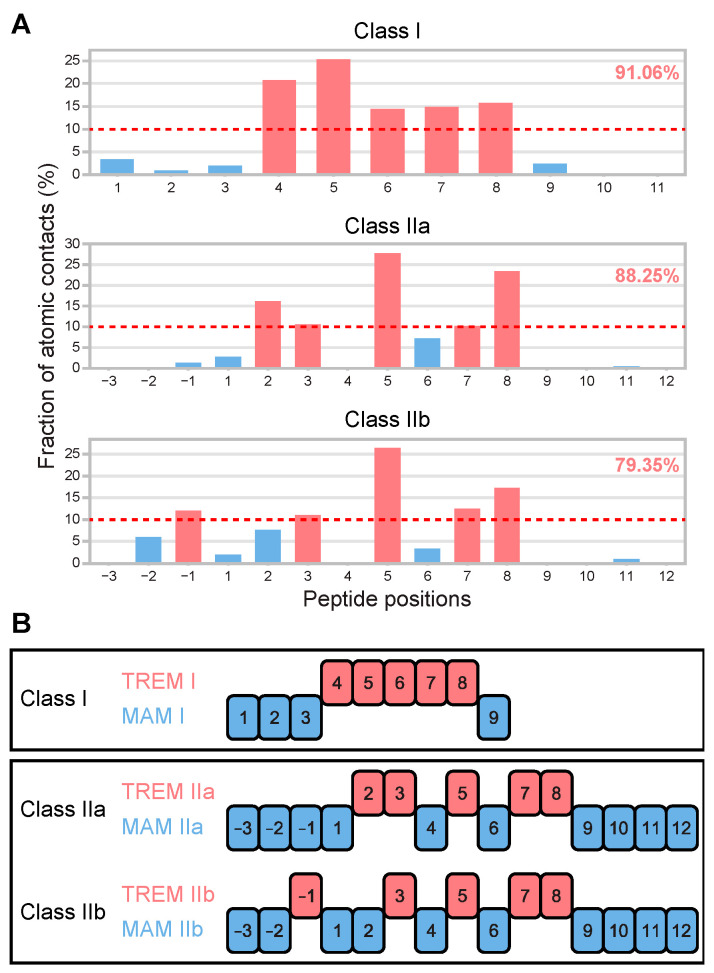
T-cell epitope motif patterns in TREM and MAM. (**A**) Fraction of atomic contacts in TCR–peptide binding was determined per position of the peptide (see Supplementary Data File S1 for details). The fraction of contacts with TCR relative to the total number of TCR–peptide binding contacts is shown with fractions of over 10%, highlighted in red, and the other positions with less contact with TCR are shown in blue. For each class of TCR–peptide binding, the total frequencies for the five positions with fractions of over 10% are indicated on the upper right. (**B**) One 9-mer class I and two 15-mer class II peptides (IIa and IIb) show distinct motif patterns in contact with TCR (TREM) and MHC (MAM). In the class I peptide, the central residues (P4–8) are recognized by the TCR, and the other positions are in contact with the MHC (MAM I). By contrast, for class II peptides, non-continuous amino acid residues in a peptide are exposed to the TCR (P2,3,5,7,8 for TREM IIa, and P-1,3,5,7,8 for TREM IIb), and the other positions show fewer contacts contributed to the interaction with MHC, such as MAM IIa and MAM IIb.

A particular subset of amino acid residues in a peptide contributes to MHC binding, and another subset of residues faces upwards to interact with the TCR [2]. To define T-cell epitope motif patterns, we comprehensively analyzed all available datasets (available in PDBj as of June 2021) of 3D structures of MHC–peptide–TCR complexes, which included 135 and 29 combinations of TCR–peptide for peptides of classes I and II peptides, respectively (Supplementary Data File S1). For peptides from both classes, certain positions in the peptide formed contact with TCR more frequently than with other positions. Five positions within each peptide constituted ≥10% of the total atomic contacts for peptides from both classes, but these positions were distinct (Figure 2). Class I peptides consistently preserved the middle positions (P4–8) of successive residues in contact with TCR across the peptides, which covered 91.06% (6907/7585) of atomic contacts. By contrast, class II T-cell epitope motifs were classified into two groups: non-continuous positions at P2, P3, P5, P7, and P8, or P-1, P3, P5, P7, and P8, in the case of 15-mer peptides. These two patterns constituted 88.25% (541/613) and 79.35% (565/712) of atomic contacts, respectively.

Hereafter, these motif patterns are termed TCR-recognized epitope motifs (TREMs) I, IIa, and IIb, and for the other MHC-binding residues, MHC agretope motifs (MAMs) I, IIa, and IIb. Because TREMs comprise pentamer amino acid combinations, each TREM theoretically represents 3.2 × 10^6^ (20^5^) combinations, and thus, the number of potential T-cell epitopes recognized by T-cells was assumed to be nearly 10 million (10^7^).

### 3.2. TREM Diversity in Germline Ig Variable Regions and the Human Proteome

T-cells are generally tolerant to germline-encoded Ig sequences [44]. Therefore, we examined the magnitude of TREMs produced from the germline sequences of the variable regions of Ig heavy chain (IGHV) fragments (Table 1). We used 336 human functional IGHV alleles and created a set of motif sequences of the three TREMs (I, IIa, and IIb) per amino acid residue position for each IGHV sequence. There were 9797 TREMs in IGHV fragments, and the number of TREMs was similar among the three TREM classes (mean, 3265.7).

To generate the T-cell population, immunological tolerance is controlled by self-antigens [45]. Given that an IGHV TREM is conserved in the human proteome, such a TREM may be excluded from a potential T-cell epitope. We, therefore, examined TREMs derived from the human proteome and the overlap between IGHV TREMs and the human proteome. The human proteome carries 7,247,747 TREMs and a mean of 2,415,916 among the three TREMs, which encompasses a large theoretical number of TREMs (75.5% of the whole set of TREMs, 2,415,916/3.2 × 10^6^), whereas only 0.1% (3265.7/3.2 × 10^6^) of the IGHV TREMs represent the theoretical number. A comparison of IGHV TREMs with the human proteome showed a substantial overlap (90% of IGHV TREMs), and only 10% were unique to IGHV TREMs (Table S1). Taken together, the magnitude and breadth of IGHV TREMs are far from encompassing the whole set of TREMs, indicating that V(D)J recombination and SHM are important for creating immunogenic idiotypic TREMs.

### 3.3. Developing a New Pipelined T-Cell Epitope Prediction Algorithm

#### 3.3.1. Pipelined Algorithm Overview

**Figure 3:**
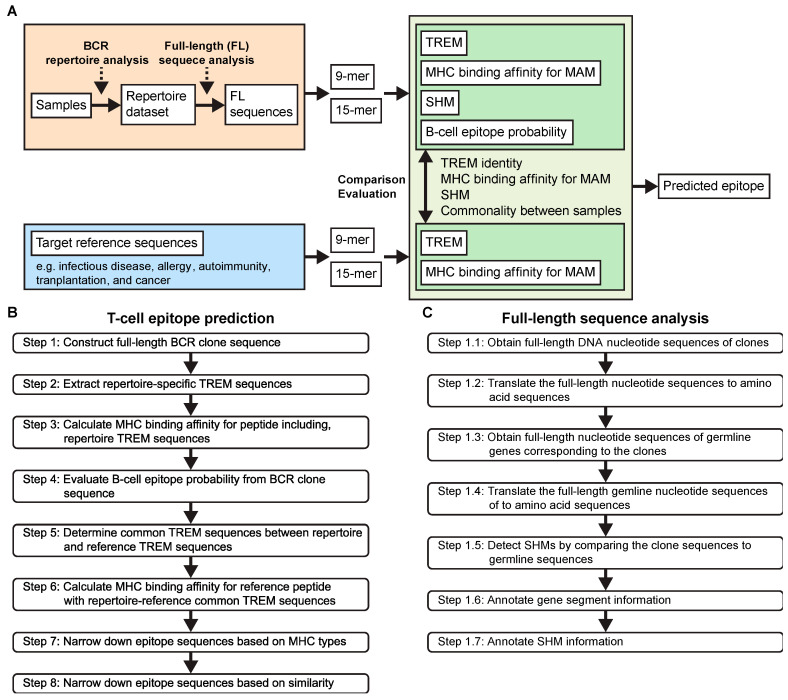
The scheme for T-cell epitope prediction. (**A**) An overview of the T-cell epitope prediction process. (**B**) A workflow of the pipeline steps of the T-cell epitope prediction process. (**C**) A workflow of the full-length sequence analysis algorithm.

The aim of our study was to develop a T-cell epitope prediction method to identify MHC-restricted, idiotypic T-cell epitope peptides from complete Ig sequences (BCR sequences), based on Jerne’s immune network theory and idiotope-driven T-B collaboration. Figure 3 displays the scheme (Figure 3A) and workflows (Figure 3B,C) of our T-cell epitope prediction method.

First, the unbiased BCR repertoire analysis method we developed [40] was employed to identify specific BCR clones (Figure 3A). Normally, the BCR repertoire observed in healthy donor samples has extremely high diversity, and BCR clones with high frequencies ≥1% are rare. By contrast, such clones are often observed in samples undergoing immune responses and are considered highly specific and related to the immunological state. Therefore, we selected such clones for subsequent analysis using our pipelined T-cell epitope prediction algorithm (Figure 3B). The FL sequences of BCR clones were produced in Step 1. Subsequently, BCR repertoire-specific TREM sequences were identified and characterized in terms of their TREM pattern, MHC binding affinity, SHM, and B-cell epitope probability in Steps 2–4. Reference TREM sequences and repertoire–reference common TREM were determined and characterized in Steps 5 and 6. After sample-specific TREM sequences were identified in Step 7, T-cell epitope candidates shared among samples were selected in Step 8.

#### 3.3.2. Full-Length Sequence Analysis (Step 1) 

To characterize a whole set of idiotopes in an Ig, the determination of the FL sequence of BCRs is vital, particularly for obtaining complete Ig sequences with point mutations caused by SHM. Therefore, we established another pipelined algorithm that reconstructed sequences obtained from repertoire analysis to produce a single FL sequence for each BCR clone, resulting in a whole set of repertoire sequences in a given sample.

FL sequence analysis involved the following seven steps (Figure 3C). At Step 1.1, the sequenced reads of each clone were obtained using the repertoire analysis data, including the VDJ gene assignment and complementarity-determining region 3 (CDR3) sequences of the clones, which were then assembled to re-construct the FL BCR gene sequences. In this step, allele information for each gene, V, D, J, and C, was also obtained from the results of repertoire analysis. At Step 1.2, the reconstructed nucleotide sequences were translated to amino acid sequences, producing FL BCR amino acid sequences. At Step 1.3, germline sequences corresponding to each BCR clone were acquired according to the allele information obtained at Step 1. At Step 1.4, the obtained germline sequences were translated to amino acid sequences, resulting in FL germline BCR amino acid sequences. At Step 1.5, SHMs were determined by comparing the FL BCR sequences with corresponding germline sequences. At Steps 1.6 and 1.7, information about the gene segments, including the definition of the framework (FR) and CDRs and the presence of SHMs, was annotated for each position on the BCR sequences.

#### 3.3.3. Extraction of the Repertoire-Specific TREM Sequences (Step 2) 

At Step 2, a set of serial 9-mer and 15-mer peptide sequences that constitute the peptides of classes I and II, respectively, was created. For each clone, both 9-mer and 15-mer amino acids were extracted from the FL BCR sequence for the target BCR clone simultaneously and its corresponding germline sequence, while sequentially shifting a single amino acid from the N-terminus to the C-terminus of the created FL BCR sequence. To create TREM and MAM motifs, we extracted 5-mer amino acid residues from the corresponding positions described in Figure 2B, and the other amino acid residues as the MAM motif. The TREM and MAM motifs were then compared between the BCR and germline sequences to determine the positions of SHMs that occurred in the BCR sequences. 

#### 3.3.4. MHC Binding Affinity Determination for TREM-Containing BCR Peptides (Step 3) 

After creating 9-mer and 15-mer peptide sequences, we calculated the IC_50_ as the MHC binding affinity for the BCR peptide sequences against MHC I and II alleles for the 9-mer and 15-mer sequences, respectively. When multiple samples were analyzed together, this analysis included a set of MHC alleles of all samples to examine whether a peptide sequence identified in a certain sample also had a binding affinity for MHC alleles of other samples.

#### 3.3.5. Linear B-Cell Epitope Probability Assessment (Step 4) 

Jerne’s idiotype network implies that an idiotypic antibody contains multiple immunogenic idiotopes, and such idiotope sequences need to be presented as continuous fragments so they can be recognized by a TCR. We thus evaluated whether the FL BCR sequences contained such linear epitope sequences, which could be targeted by antibodies. For each clone, the FL BCR sequences were analyzed using BepiPred-2.0. When 9-mer or 15-mer peptide sequences overlapped any amino acids with a >0.5 score, they were considered B-cell linear epitope sequences and denoted as “E”. However, known B-cell epitope prediction methods are still unreliable [46]. Therefore, even when epitope sequences did not show linear B-cell epitope probability, they were not excluded from epitope selection.

#### 3.3.6. Molecular Mimicry in BCR and Antigen Reference Sequences (Step 5) 

To search for molecular mimicry in BCR sequences, we compared repertoire-specific TREMs with antigen reference sequences and examined whether the same TREMs existed in the reference sequences. If a TREM was conserved between the BCR and reference sequences, the TREM was confirmed as a repertoire–reference common TREM, with possible molecular mimicry. For conserved TREMs, 9-mer or 15-mer peptide sequences including TREMs were extracted from reference sequences.

#### 3.3.7. MHC Binding Affinity Determination of the Reference Peptide Sequences (Step 6) 

Once collected from the reference TREM sequences, reference peptide sequences containing repertoire–reference common TREMs were analyzed for MHC binding affinity using the same MHC alleles as those used for the BCR sequences in Step 3, and the IC_50_ values were determined for each reference peptide sequence.

#### 3.3.8. Epitope Sequence Refinement Based on MHC Types (Step 7) 

We refined the list of epitope sequences based on HLA types and IC_50_ values. For each repertoire–reference common TREM, a pair of sequences comprising a repertoire-specific peptide sequence and reference peptide sequence was examined to determine whether the IC_50_ values of both sequences were below a defined threshold (5000 by default) against HLA alleles of the sample. The resulting TREMs were confirmed as sample-specific TREMs, and the peptide sequences containing TREMs were predicted to be T-cell epitopes of the sample.

#### 3.3.9. Narrowing down Results Based on Similarity (Step 8) 

After performing Steps 1–7 for all samples, we evaluated the TREMs and corresponding peptides in terms of conservation and versatility. TREMs shared by more than two samples were considered conserved TREMs, whereas TREMs present only in one sample were sample-specific, unique TREMs. To assess versatility, peptides containing each TREM were examined in terms of the frequency and strength of their binding to different MHC alleles using all available MHC alleles and different thresholds of IC_50_ values. Peptides with the potential to strongly interact with many MHC alleles were considered as having high versatility, ranging from + to +++; + for peptides capable of binding to at least one MHC allele at an IC_50_ of 5000 or lower, ++ for those at an IC_50_ of 500 or lower, and +++ for those at an IC_50_ of 50 or lower. The more common the TREM motif, the higher the likelihood it would be recognized by the immune systems of different individuals.

### 3.4. T-Cell Epitope Prediction in DENV Infection

DENV infection is a mosquito-borne flavivirus infection [47]. There are four serotypes of DENV (DENV-1 to -4). A major challenge in DENV infection is the occurrence of secondary infection with a different serotype from the primary infection, which may lead to severe clinical manifestations, such as dengue hemorrhagic fever or dengue shock syndrome, and effective vaccines against DENV are still lacking.

To assess whether our T-cell epitope prediction method could identify T-cell epitopes that induce T-cell responses, we used 45 PBMC samples collected at the acute phase of disease and during two convalescent phases (at 8 months and 1 year) after DENV infection from 18 individuals who had primary (*n* = 8) or secondary (*n* = 10) DENV infection [26,27]. Most individuals were infected with serotype DENV-1 (*n* = 15), but a few were infected with DENV-2 (*n* = 3). It was confirmed that all patients exhibited detectable levels of neutralizing activity against these serotypes, as well as some other serotypes at the acute and convalescent phases.

**Figure 4:**
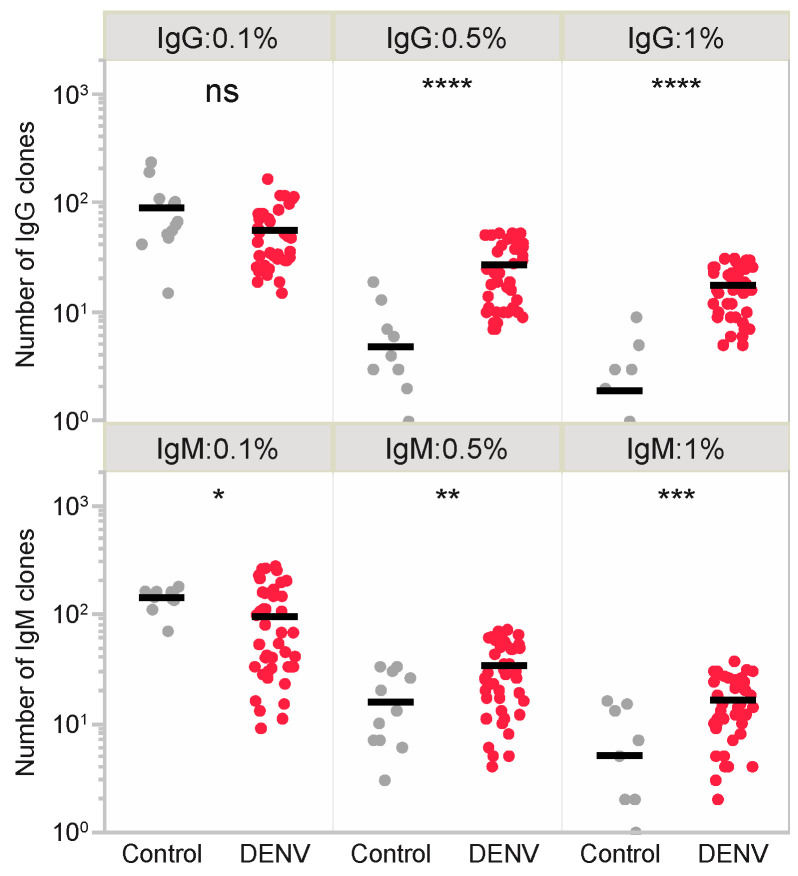
Expansion of IgG and IgM clones in response to DENV infection. The number of BCR clones (IgG, upper and IgM, lower) were compared between healthy donor controls and DENV infection for each of the frequencies over 0.1%, 0.5%, and 1%. Black bars indicate the mean number of clones. Statistical significance was analyzed using the Mann–Whitney U test. * *p* < 0.05, ** *p* < 0.01, *** *p* < 0.001, **** *p* < 0.0001, ns: not significant.

Using these samples, we performed BCR repertoire analysis directed against IgG and IgM. The BCR repertoire analysis revealed a strong skewing of IgG and IgM repertoires with high frequencies of BCR clones (Figure 4). The total number of BCR clones with a frequency ≥1% was 1427 (725 for IgG and 702 for IgM) (Table 2), and this was significantly higher in DENV samples, including those from the acute and convalescent phases, compared with that in healthy donor controls with no DENV infection history (only 84 clones; 23 for IgG and 61 for IgM) (Figure 4 and Figure S1). The expansion of these BCR clones was considered highly related to DENV infection, and these clones were used for subsequent analysis to predict DENV-specific T-cell epitopes. Importantly, none of the BCR clones overlapped among the samples, even during different phases within the same individual.

**Figure 5:**
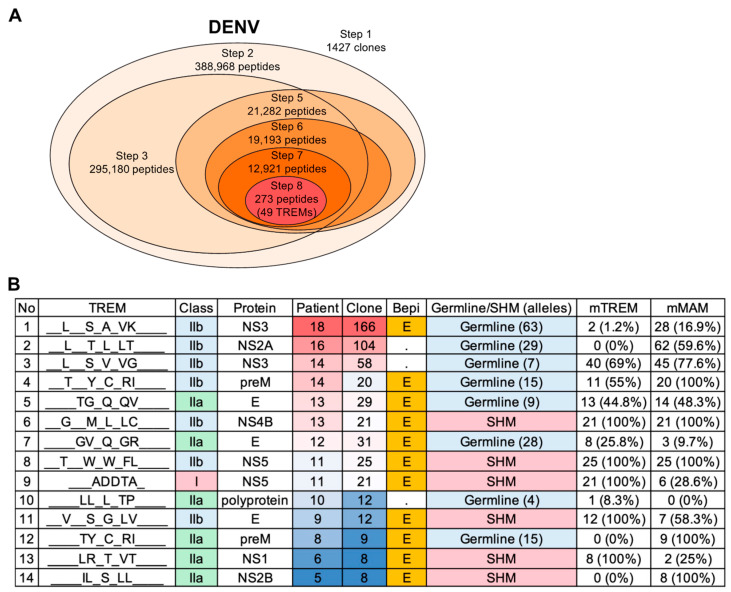
Predicted TREMs in DENV infection. (**A**) Summary of the number of peptides resulting from each step of the epitope prediction for DENV. The number of clones or peptides includes all samples examined, and peptides were selected and narrowed down using Vietnam isolates as primary reference sequences at Step 8. A total of 49 TREMs were identified by analyzing 1427 clones with a frequency of ≥1% from 18 DENV patients. (**B**) Top 14 TREMs shared by more than five individuals (see also Supplementary Data File S2 for details of all 49 TREMs). Among them, 46 TREMs were class II epitopes (IIa or IIb). TREMs shared among individuals tend to germline-typed sequences, whereas patient-unique TREMs contain SHMs and are found in CDR3 overlapping regions. A total of 43 out of 49 showed B-cell linear epitope potential. A total of 22 TREMs were found in T-cell epitopes that have previously been reported to induce T-cell activity (Supplementary Data File S2). Note that SHMs are also found in MAM sequences.

To identify repertoire–reference common TREMs, DENV-1 isolates determined in the donors were used as primary reference sequences. The analysis also included all other DENV serotypes (DENV-1 to -4) and other flaviviruses as reference sequences to examine possible cross-reactivity. HLA binding was predicted against the HLA alleles of each donor. Epitope prediction identified 49 TREM epitopes present in the DENV-responsive BCR sequences and DENV-1 proteomes (Table 2, Figure 5A). Of the clones, 29.5% (421/1427) possessed the TREMs in their BCR sequences. On average, 30.6% of clones carried at least one TREM, and so not all clones contained the identified TREMs. Of the TREM-containing clones, 50.4% possessed SHMs in the TREM sequences (SHM TREMs). The BCR clones predominantly contained DENV-related TREMs. Despite a larger number of clones, the analysis of 2566 BCR clones with a frequency of ≥0.5% resulted in only 14 more TREMs, and 4 additional TREMs for 6417 clones with a frequency of ≥0.1%.

In agreement with the role of B-cells as professional APCs, most of the identified TREMs (93.9%, 46/49) were class II epitopes (Figure 5B, Supplementary Data File S2). The identified TREMs were present in structural proteins, such as envelope (E) and membrane (M) proteins, and non-structural proteins (NSs) 1–5. In addition to DENV-1, TREMs were also conserved in other DENV serotypes (57.1%, 28/49) and other flaviviruses, including YFV, WNV, JEV, ZIKA, and TBEV (36.7%, 18/49) (Supplementary Data File S2), consistent with the previously reported potential cross-reactivity among these viruses [48]. A number of TREMs (85.7%, 42/49) also shared identical TREM patterns with the human proteome (Supplementary Data File S2). Despite there being no overlap in BCR clones among the individuals, half of the TREMs (24/49) were shared among patients, and the other TREMs were unique to individuals. Importantly, 87.8% (43/49) of TREMs contained B-cell linear epitope potential sequences (Figure 5B, Supplementary Data File S2).

### 3.5. SHM Contributes to Diversity in Epitope Patterns and Influences HLA Binding

**Figure 6:**
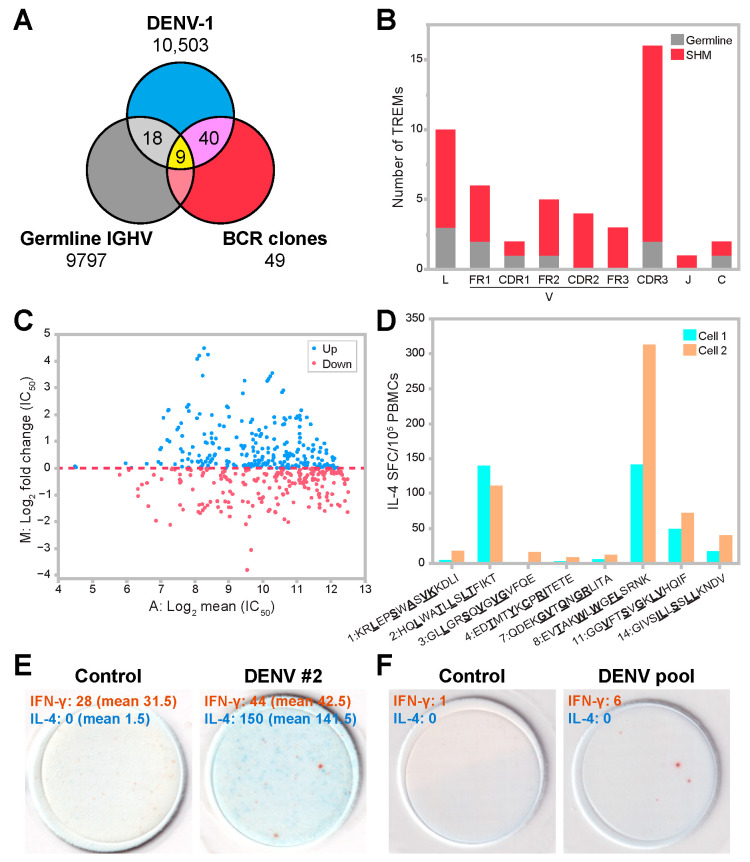
Characterization and T-cell immunogenicity of TREM/MAM epitopes from DENV-responsive BCR clones. (**A**) Overlap of TREM sequences among DENV-1, germline IGHV, and DENV-responsive BCR clones. (**B**) Distribution of identified TREMs in the variable region of the immunoglobulin sequence. (**C**) MA plot showing alternation of MHC binding affinity (IC_50_) from germline MAM sequences to mutated sequences in BCR clones. M: log_2_(IC_50_ of BCR peptide sequences with MAM mutated/IC_50_ of germline BCR peptide sequences). A: 1/2(log_2_(IC_50_ of BCR peptide sequences with MAM mutated) and log_2_(IC_50_ of germline BCR peptide sequences). Blue (up) and red (down) points indicate increased and reduced HLA binding affinity by SHM, respectively. (**D**) IL-4-producing spot-forming cell (SFC) counts following stimulation with eight different peptides in cultured ELISPOT assays. Details of the peptides are shown in Table 3. PBMCs derived from two different donors were tested. Peptide sequences with TREM in bold and underlined text are indicated. (**E**) Representative cultured ELISPOT assays of T-cell responses with control (left) and DENV no. 2 peptide (HQLWATLLSLTFIKT, right). The representative images and spot counts are shown for IFN-γ and IL-4, with the mean number of spots of duplicate wells shown in parenthesis. (**F**) Ex vivo ELISPOT assays showing T-cell stimulation with a pool of eight identified peptides in PBMCs from cases with a history of DENV infection (right) but not in DMSO controls (left). The representative images and spot counts are shown for IFN-γ and IL-4.

We investigated whether SHMs that occur in BCR sequences have any impact on T-cell epitope features. The patient-derived DENV-1 proteomes contained 10,503 TREMs, and only 27 of these overlapped with germline IGHV TREMs (12 TREM I, 7 TREM IIa, and 8 TREM IIb) (Figure 6A, Table S2). The 49 identified TREMs contained 9 TREMs from the IGHV-DENV-1 conserved TREMs and 1 IGHC-DENV-1 conserved TREM. The other 39 TREMs (79.6%) included mutations and did not exist in germline Ig sequences. Thus, SHMs effectively contributed to the generation of antigen-shared TREMs.

Although germline and SHM TREMs were distributed across the variable region, SHM TREMs were most frequently found in CDR3-containing regions (32.7%, 16/49) (Figure 6B). This analysis showed that germline TREMs were shared among patients, whereas SHM TREMs tended to be patient-specific. However, there were 113 cases in which mutations occurred independently in the BCR clones of different patients resulting in 14 patterns of the same SHM TREMs (Supplementary Data File S2). There was also a substantial number of cases in which SHMs converted BCR sequences into IGHV-DENV-1 conserved germline TREMs (13.4%, 78/583 clones).

Epitope prediction revealed that MAMs contained SHMs in a significant number of cases (49.9%, 291/583) (Figure 5B, Supplementary Data File S2). Because previous work suggested that mutations in idiotope-derived peptides influenced MHC binding [18], we examined the effect of SHMs in MAM sequences for MHC binding. This analysis showed that 24.3% of MAMs gained higher binding affinity against patient-typed HLA alleles (Figure 6C). Thus, SHMs in 24.3% of MAMs might enhance the selection and expansion of BCR clones by presenting idiotype-derived peptides with an elevated affinity for a particular individual’s HLA molecules to idiotope-specific T-cells.

### 3.6. Predicted TREM Epitopes Exhibit T-Cell Immunogenicity

We next investigated the potential T-cell immunogenicity of the identified TREM epitopes. A database screen of the IEDB (https://www.iedb.org, accessed on 10 May 2023) confirmed that 22 TREMs (44.9%) overlapped with DENV T-cell epitopes that had been reported to induce T-cell activation (Supplementary Data File S2). To further evaluate the predicted epitopes for T-cell reactivity, we selected the identified TREMs that were shared by more than five individuals and then further refined the list of epitope candidates by taking those that had relatively high versatility (++ or +++), using the versatility criteria defined above (Step 8), which resulted in eight DENV peptide sequences containing the identified TREMs that were shared by more than five patients (Table 3). These included epitopes with and without previous reports of T-cell activity and both germline- and SHM-derived epitopes, all of which contained MAM mutations in the corresponding BCR sequences.

To examine whether the selected epitopes induce T-cell responses, we tested two sets of PBMCs derived from healthy donors with different HLA types and from those without DENV infection in cultured ELISPOT assays. PBMCs were stimulated with each of the eight peptides individually, and the spots of cells producing IFN-γ and IL-4 were counted. Both PBMCs showed similar responses: IL-4 production was observed, but IFN-γ gave high background responses. We thus concluded that IFN-γ production reflected mostly non-specific responses in the cultured ELISPOT assays. Half of the peptides (peptides 2, 8, 11, and 14), which had stronger HLA binding affinity for the tested HLA alleles (Table 3 and Table S3), exhibited notable T-cell immunogenicity (Figure 6D,E). Among the confirmed peptides were all the peptides containing SHMs in the TREM sequences and only one germline-derived TREM peptide, suggesting that SHMs are more effective at providing T-cell immunogenicity. To further confirm the T-cell immunogenicity of the peptides, we performed ex vivo ELISPOT assays using PBMCs from an individual with a history of DENV infection. Stimulation with the pooled peptides elicited T-cell responses (Figure 6F). The responses might also include the cross-reactivity of T-cells because some epitopes are conserved between different DENV serotypes and other flaviviruses. Such conserved epitopes could induce protective immune responses, and thus, be vaccine candidate sequences. Taken together, our T-cell epitope prediction method efficiently identified T-cell stimulatory epitopes.

### 3.7. Validation of the T-Cell Epitope Prediction Method for COVID-19

SARS-CoV-2 is the causative agent of coronavirus disease 2019 (COVID-19) and is responsible for the current worldwide pandemic [49]. Toward understanding the human T-cell immunity and pathogenesis caused by SARS-CoV-2, as well as developing effective vaccines, a number of epitope predictions against T-cell stimulating epitopes were made [50]. Such studies identified SARS-CoV-2-specific epitopes and conserved epitopes across other coronaviruses, totaling over 58,000 epitopes as of January, 2022, in the IEDB. Given such deposition of epitopes, SARS-CoV-2 provides a great opportunity to assess our T-cell epitope prediction method further.

**Figure 7:**
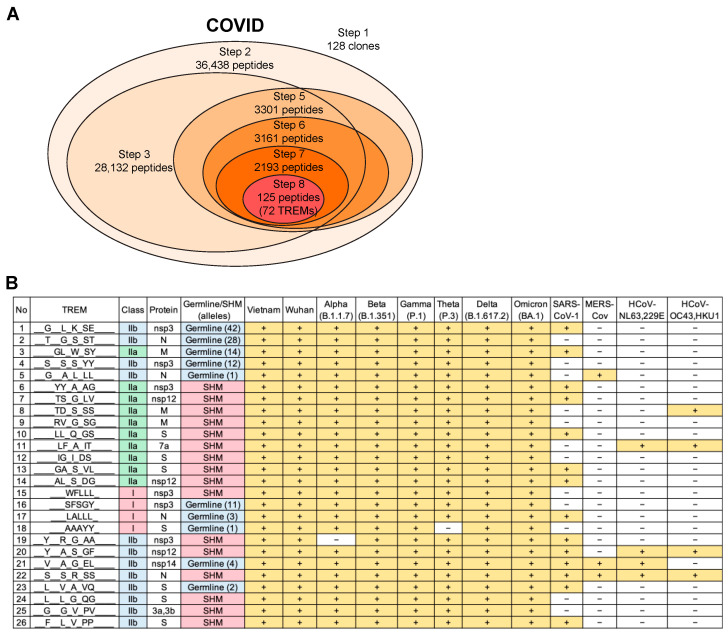
Predicted SARS-CoV-2 T-cell epitopes with previously confirmed T-cell activity. (**A**) Summary of the number of peptides resulting from each step of the epitope prediction for COVID. The number of clones or peptides include all samples examined, and peptides were selected and narrowed down using Vietnam isolates as primary reference sequences at Step 8. (**B**) A total of 26 out of 72 TREMs that were identified by analyzing 128 clones with a frequency ≥1% from 20 COVID-19 patients were among the T-cell epitopes that had previously been reported to induce T-cell activity (see also Supplementary Data File S3 for details), and conserved across SARS-CoV-2 variants and other coronaviruses (denoted as “+”).

We predicted SARS-CoV-2 T-cell epitopes using PBMCs isolated from 20 COVID-19 patients from Vietnam who tested positive for SARS-CoV-2 infection [28]. BCR repertoire analysis resulted in 128 BCR clones with a frequency of ≥1% (98 for IgG and 30 for IgM). As in DENV infection, there were no overlapping clones among patients, and thus, it was confirmed that disease-specific BCR clones were highly specific to individuals. T-cell epitope prediction was conducted using these clones and reference sequences, including three SARS-CoV-2 isolates that were collected in Vietnam, the original strain from Wuhan, other SARS-CoV-2 variants B.1.1.7, B.1.351, P.1, P.3, B.1.617.2, and BA.1, and other coronaviruses, such as SARS-CoV-1, Middle East respiratory syndrome coronavirus (MERS-CoV), and common cold coronaviruses (CCCs) NL63, 229E, OC43, and HKU1. Our analysis revealed 72 SARS-CoV-2 TREMs (Figure 7A), of which 26 (36.1%) were found in the reported SARS-CoV-2 T-cell epitopes with T-cell activity, such as cytokine release, activation, cytotoxicity, qualitative binding, and proliferation (Figure 7B, Supplementary Data File S3). The corresponding proteins included spike (S), open reading frame 3a (ORF3a), ORF3b, ORF7a, membrane (M), nucleocapsid (N), non-structural protein 3 (nsp3), nsp12, and nsp14. Among the remaining 46 TREMs, 8 (11.1%) were reported to be negative for T-cell reactivity, and it had not been studied whether the other 38 (52.8%) could stimulate T-cells. Therefore, more positive TREMs were included in the predicted epitopes.

As for the DENV epitopes, the SARS-CoV-2 TREMs included SHM IGHV TREMs, germline IGHV TREMs, and matched human proteome-derived TREMs. Because these TREMs were previously confirmed to be T-cell-stimulating epitopes, the data support the notion that germline IGHV TREMs and TREMs encoded in the human genome do not simply provide tolerance to T-cells.

All 26 identified TREMs we examined were conserved across all SARS-CoV-2 variants, and several were also shared with SARS-CoV-1, MERS-CoV, and CCCs (Figure 7B), which may relate to previous findings of cross-reactive T-cell immunity [51]. Taken together, these results demonstrated that our T-cell epitope prediction algorithm enabled the identification of functional T-cell epitopes recognized by the T-cells that stimulate individual-specific and patient-common immune responses in infectious diseases.

## 4. Discussion

T-cell epitope prediction is important for understanding T-cell immunity in various diseases and for developing vaccines. Many prediction tools focus on peptide binding to MHC molecules because of their role in antigen presentation. By contrast, antigen recognition by TCRs has not received the same attention, and thus, remains to be explored despite its equal importance in terms of T-cell recognition. Therefore, in the present study, we determined T-cell epitope motif patterns, which we defined as “TREM” for amino acid residues at particular positions that interact with TCR molecules and “MAM” for other amino acid residues binding to MHC molecules. Similar patterns were observed previously [52,53,54].

Our T-cell epitope prediction method was based on the following hypotheses. First, an antigen-specific antibody-carrying B-cell presents antigen peptides, and antigen-specific cognate T-cells aid the B-cell proliferation and differentiation. Second, given the molecular mimicry in idiotope sequences in anti-idiotypic antibodies of a second B-cell, the idiotope peptide would be presented via idiotope-driven T-B collaboration, where the antigen-specific T-cells also recognize it and provide the same help for the second B-cell, because the T-cells cannot discriminate between the “TREMs” presented by the two B-cells. Consequently, the idiotope-producing B-cells become abundant and their BCR sequences are detected by repertoire analysis. By analyzing such abundant BCR clones, we identified TREM epitopes with molecular mimicry for antigens in the cases of two different infectious diseases caused by DENV and SARS-CoV-2 infection. In contrast, there were no identical 9-mer or 15-mer sequences between BCR and the antigens, which confirms that TREM motifs have more relevance for T-cell epitope prediction than the complete peptide sequences. The identified epitopes were validated in this study, and some were confirmed by previous reports.

Interestingly, despite there being no overlap in the BCR clones, our analysis revealed the conservation of TREMs among individuals and among distinct clones during different phases in the same individual, with the maximum observed conservation being all 18 individuals in DENV infection. This indicates that even though every BCR clone is unique, the BCR repertoire appears to converge among different individuals experiencing viral infection, and changes over time, while maintaining a certain degree of antigen specificity. The conserved TREMs may represent such a factor, being involved in this type of convergence and maintenance. The conserved TREMs included many germline TREMs. Although it was previously shown that T-cells are tolerant to germline-encoded Ig sequences [44], our results demonstrated the T-cell immunogenicity of the germline TREM. This germline TREM had SHMs in MAM sequences in 59.6% of clones, and 24.3% sequences with MAMs mutations showed increased binding affinity. Thus, unlike previous data, germline TREMs would not readily provide T-cell tolerance, and T-cell tolerance to germline BCR sequences could depend on individual HLA types, rather than the sequence itself. This may also be true for human proteome TREMs [55] because TREMs overlapping with the human proteome were among the confirmed TREMs for T-cell stimulation.

In addition to affecting MAM sequences, SHMs and V(D)J recombination, as expected, appeared more important for controlling TREM sequences in terms of T-cell tolerance and T-cell immunogenicity because of the following reasons. First, most of the identified reference–repertoire common TREMs were SHM TREMs and were present in CDR3-containing regions. Second, SHMs converted germline BCR sequences to antigen-specific TREMs. Third, T-cell immunogenicity was confirmed for all tested SHM TREMs (3/3), unlike germline TREMs (1/5). Furthermore, SHMs and V(D)J recombination might facilitate the creation of individual-specific and public T-cell populations. The patient-specific, unique TREMs were almost all SHM TREMs. By contrast, there were convergent mutations that converted one germline IGHV allele sequence into another germline sequence in different individuals, which resulted in the same conserved TREMs. These conserved TREMs may be prominent T-cell epitopes that are associated with antigen specificity and are commonly recognized by T-cells in various individuals.

Recently, it has been reported that autoantibodies against the angiotensin-converting enzyme 2 receptor, a binding target of the SARS-CoV-2 S protein, are generated after SARS-CoV-2 infection, and this may have implications for the development of long COVID after SARS-CoV-2 infection [56,57]. These antibodies represent anti-idiotypic antibodies that have molecular mimicry with S proteins. Furthermore, autoantibodies against chemokines were observed in COVID-19 and are associated with severity and long COVID [58]. These autoantibodies may induce anti-idiotypic antibodies that mimic those chemokines, or might be anti-idiotypic antibodies that have sequences resembling their receptors. These provide evidence that anti-idiotypic antibodies are induced in infectious diseases, and strongly supports our concept of T-cell epitope prediction, which involves searching for mirror images of antigens in BCR sequences. Therefore, our T-cell epitope prediction strategy will also be useful for investigating the cause of such autoimmunity that results from viral infection, as well as diseases involving changes in immunological status.

In general, it is thought that antibodies target structural epitopes, whereas TCRs recognize linear epitopes. Thus, the molecular mimicry of anti-idiotypic antibodies may represent such a “structural” property. However, TREM-like linear epitopes repeatedly found in antibodies stimulated the production of antibodies against the original antigens, and elicited T-cell responses [12]. Despite these findings, there is still a substantial gap in our knowledge regarding the extent to which T-cell linear epitopes with molecular mimicry are present in BCR sequences. Further comprehensive identification and experimental examination of T-cell epitopes in BCRs are needed to understand the importance of the T/B-shared linear epitopes with molecular mimicry in the immune system.

## 5. Conclusions

Here, we developed a T-cell epitope prediction method by combining the concepts of the idiotype network theory and idiotope-driven T-B collaboration. This prediction method was applied to two cases of infectious disease, DENV and SARS-CoV-2, but it will also be applicable to other immune responses, where idiotope-driven T-B collaboration is involved.

## Figures and Tables

**Table 1:** Composition of IGHV TREMs.

No. of Alleles	TREM I (3258)	TREM IIa (3251)	TREM IIb (3288)	Average (3265.7)
1 (0.3%)	33.6% (1095)	33.7% (1097)	33.8% (1111)	33.7% (1101)
2–5 (0.6–1.49%)	35% (1140)	34.5% (1123)	34.4% (1132)	34.7% (1131.7)
6–9 (1.79–2.68%)	9.6% (314)	10.5% (340)	10.3% (340)	10.1% (331.3)
10–20 (2.98–5.95%)	9.8% (318)	9.4% (306)	9.3% (307)	9.5% (310.3)
21–41 (6.25–12.5%)	5.2% (171)	5.5% (179)	5.8% (191)	5.5% (180.3)
42–83 (12.5–25%)	4.2% (138)	4% (131)	4.3% (141)	4.2% (136.7)
84–167 (25–50%)	2.2% (72)	2% (66)	1.9% (61)	2% (66.3)
168+ (>50%)	0.3% (10)	0.3% (9)	0.2% (5)	0.2% (8)

**Table 2:** High frequency DENV-1-responsive BCR clones with a TREM and SHM.

Types	IgG	IgM	IgG and IgM
≥1% clones ^a^	725 (5–31)	702 (2–37)	1427 (2–37)
TREMs	29	37	49
TREM clones ^a^	129 (0–12)	292 (0–17)	421 (0–17)
SHM clones ^a^	77 (0–9)	111 (0–7)	188 (0–9)
TREM clones in ≥1% clones (%) ^b^	18.1 (0–57.1)	42.8 (0–100)	30.6 (0–100)
SHM clones in ≥1% clones (%) ^c^	10.6 (0–57.1)	16.5 (0–66.7)	13.6 (0–66.7)
SHM clones in TREM clones (%) ^d^	61.9 (0–100)	40.4 (0–100)	50.4 (0–100)
Maximum TREM ^e^	1.8 (0–5)	1.7 (0–4)	1.8 (0–5)
Maximum SHM ^e^	1 (0–4)	1 (0–2)	1 (0–4)

^a^ Total number of clones with ≥1% frequency. TREM and SHM clones indicate TREM-containing clones and SHM-containing TREM clones among the ≥1% high-frequency clones, respectively. Parentheses show the range of the number of clones for all samples. ^b^ Average percentage of the number of TREM clones among the ≥1% high-frequency clones per sample. Parentheses show the range of the number of clones for all samples. ^c^ Average percentage of the number of SHM clones among the ≥1% high-frequency clones per sample. Parentheses show the range of the number of clones for all samples. ^d^ Average percentage of the number of SHM clones among the TREM clones per sample. Parentheses show the range of the number of clones for all samples. ^e^ Average maximum number of TREMs or SHM-containing TREMs per clone. Parentheses show the range of the number of TREMs for all samples.

**Table 3:** DENV-1-derived T-cell epitope peptides tested in cultured ELISPOT assays.

No	TREM	Germline/ SHM	Protein	Epitope Sequence	Versatility ^a^	Cell 1 IC_50_ Min ^b^	Cell 2 IC_50_ Min ^b^	Cultured ELISPOT Assays
1	__L__S_A_VK____	Germline	NS3	KRLEPSWASVKKDLI	++	266.82	530.23	no
2	__L__T_L_LT____	Germline	NS2A	HQLWATLLSLTFIKT	+++	37.97	56.26	yes
3	__L__S_V_VG____	Germline	NS3	GLLGRSQVGVGVFQE	++	884.5	390.99	no
4	__T__Y_C_RI____	Germline	preM	EDTMTYKCPRITETE	++	2496.2	1098.35	no
7	____GV_Q_GR____	Germline	E	QDEKGVTQNGRLITA	++	1571.02	376.13	no
8	__T__W_W_FL____	SHM	NS5	EVTAKWLWGFLSRNK	+++	122.72	290.34	yes
11	__V__S_G_LV____	SHM	E	GGVFTSVGKLVHQIF	+++	50.14	77.93	yes
14	____IL_S_LL____	SHM	NS2B	GIVSILLSSLLKNDV	+++	92.23	80.6	yes

^a^ Peptides capable for binding to at least one MHC allele at an IC_50_ of 500 or lower are indicated by ++, and those at an IC_50_ of 50 or lower by +++. ^b^ Minimum values of HLA binding affinity among all alleles (see Table S3 for details).

## Data Availability

The data presented in this study are available in this article.

## Supplementary Materials

The following supporting information can be downloaded at: https://www.mdpi.com/article/10.3390/v15051186/s1, Figure S1: Expansion of IgG and IgM clones in response to DENV infection persists during the convalescent phases; Table S1: Distribution and conservation of IGHV TREMs with the human proteome; Table S2: Conserved TREMs between germline Ig sequences and DENV-1; Table S3: HLA binding affinity of PBMCs tested in cultured ELISPOT assays; Supplementary Data File S1: TCR–peptide–MHC structure analysis; Supplementary Data File S2: 49 predicted TREMs in DENV infection; Supplementary Data File S3: 26 predicted SARS-CoV-2 T-cell epitopes with previously confirmed T-cell activity.
