# Supplementary material for: Idiotope-Driven T-Cell/B-Cell Collaboration-Based T-Cell Epitope Prediction Using B-Cell Receptor Repertoire Sequences in Infectious Diseases
Source: Viruses. 2023 May 17;15(5):1186. doi: 10.3390/v15051186 (PMC10221809; doi:10.3390/v15051186)
Supplement: Supplementary file 1 [file viruses-15-01186-s001.zip › Supplementary_Materials.pdf]

Supplementary Materials

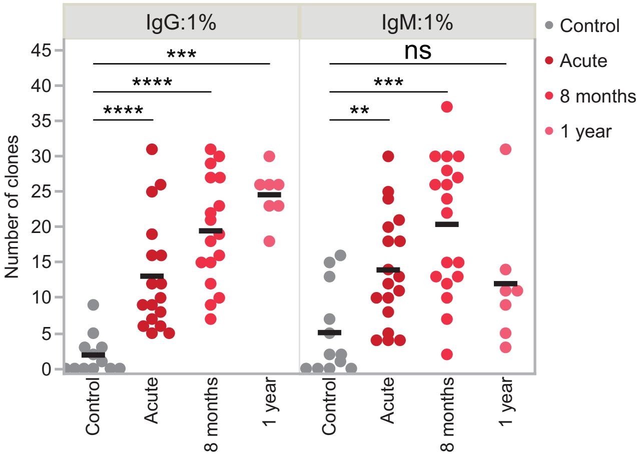

Figure S1. Expansion of IgG and IgM clones in response to DENV infection persists during the convalescent phases. The number of BCR clones (IgG and IgM) with a frequency of over 1% were compared between healthy donor controls and the different phases of DENV infection. Expanded BCR clones persist for 8 months (IgG and IgM) and even 1 year (IgG) after infection. Black bars indicate the mean number of clones. Statistical significance was analyzed using the Mann–Whitney U test. $^{**}p < 0.01$, $^{***}p < 0.001$, $^{****}p < 0.0001$, ns: not significant.

Table S1. Distribution and conservation of IGHV TREMs with the human proteome.

Region [a]	IGHV				Unique in IGHV				Overlap with human proteome			
	TREM I	TREM IIa	TREM IIb	mean	TREM I	TREM IIa	TREM IIb	mean	TREM I	TREM IIa	TREM IIb	mean
Total	100% (3272)	100% (3275)	100% (3314)	100% (3287)	10.8% (354)	9.4% (309)	9.7% (323)	10% (328.7)	89.2% (2918)	90.6% (2966)	90.3% (2991)	90% (2958.3)
L	12.8% (418)	13.3% (437)	13.5% (448)	13.2% (434.3)	0.6% (19)	0.3% (9)	0.5% (15)	0.4% (14.3)	12.2% (399)	13.1% (428)	13.1% (433)	12.8% (420)
FR1	19.3% (633)	21.8% (715)	21.2% (701)	20.8% (683)	0.3% (9)	1.3% (43)	1.4% (46)	1% (32.7)	19.1% (624)	20.5% (672)	19.8% (655)	19.8% (650.3)
CDR1	12.1% (395)	9.7% (317)	10% (331)	10.6% (347.7)	4.8% (157)	3.1% (100)	2.4% (80)	3.4% (112.3)	7.3% (238)	6.6% (217)	7.6% (251)	7.2% (235.3)
FR2	19.1% (625)	21.4% (702)	21.1% (699)	20.5% (675.3)	2.4% (78)	2.8% (92)	2.8% (93)	2.7% (87.7)	16.7% (547)	18.6% (610)	18.3% (606)	17.9% (587.7)
CDR2	10.8% (353)	9.5% (310)	9.7% (323)	10% (328.7)	1.1% (37)	0.5% (17)	0.7% (24)	0.8% (26)	9.7% (316)	8.9% (293)	9% (299)	9.2% (302.7)
FR3	25.9% (848)	24.2% (794)	24.5% (812)	24.9% (818)	1.7% (54)	1.5% (48)	2% (65)	1.7% (55.7)	24.3% (794)	22.8% (746)	22.5% (747)	23.2% (762.3)

[a] TREMs that are located across two regions were counted twice.

Table S2. Conserved TREMs between germline Ig sequences and DENV-1.

	TREM I	TREM IIa	TREM IIb
IGHV	___AISGD_	____GV_Q_GR____ [a]	__E__T_G_TT____
	___GPSLR_	____LL_L_TP____ [a]	__G__G_A_GS____
	___IFGTA_	____RL_S_VR____	__G__L_W_GL____ [a]
	___KPGTS_	____SR_T_SI____	__L__A_A_GT____
	___LLLTI_	____TG_Q_QV____ [a]	__L__S_A_VK____ [a]
	___NSRNT_	____TY_C_RI____ [a]	__L__S_V_VG____ [a]
	___SGKGL_	____VY_G_IS____	__L__T_L_LT____ [a]
	___SGRGL_		__T__Y_C_RI____ [a]
	___SGVSW_		
	___SWNSG_		
	___VKPSG_		
	___YVSAI_		
IGHC	___KEKEE_	____DI_V_LL____	__I__S_A_IF____
	___SRSTS_ [a]	____LP_S_EE____	__P__A_L_KS____
	___SWNSG_	____NE_V_LT____	__R__G_A_KT____
	___WNTGE_	____RE_K_VG____	__S__Q_G_IF____
		____SG_Y_LS____	__T__T_P_TT____
		____TA_L_KS____	__V__F_L_LL____
		____TQ_E_TL____	

[a] TREMs found as DENV-1 T-cell epitopes in BCR clones.

Table S3. HLA binding affinity of PBMCs tested in cultured ELISPOT assays.

	No	1	2	3	4	7	8	11	14
	TREM	__L__S_A_VK____	__L__T_L_LT____	__L__S_V_VG____	__T__Y_C_RI____	____GV_Q_GR____	__T__W_W_FL____	__V__S_G_LV____	____IL_S_LL____
	Protein	NS3	NS2A	NS3	preM	E	NS5	E	NS2B
	Epitope sequence	KRLEPSWASVKKDLI	HQLWATLLSLTFIKT	GLLGRSQVGVGVFQE	EDTMTYKCPRITETE	QDEKGVTQNGRLITA	EVTAKWLWGFLSRNK	GGVFTSVGKLVHQIF	GIVSILLSSLLKNDV
	Position	1538-1552	1270-1284	1504-1518	160-174	620-634	2867-2881	706-720	1357-1371
Cell 1	DPA1*01:03/DPB1*04:01	5346.66	56.26	5351.99	12281.74	14299.56	402.82	1178.63	160.24
	DPA1*01:03/DPB1*05:01	2773.03	317.85	4503.13	8850.55	9421.11	581.88	707.39	277.91
	DPA1*02:02/DPB1*04:01	2543.42	37.97	3817.02	6625.83	8947.99	258.08	686.41	92.23
	DPA1*02:02/DPB1*05:01	2578.31	151.87	5882.87	8511.89	8751.33	504.5	549.42	132.78
	DQA1*01:02/DQB1*03:03	3659.1	1679.17	2580.79	10093.66	5262.41	5165.72	2669.29	2032.27
	DQA1*01:02/DQB1*05:02	6242.49	1959.84	6065.85	11018.07	16466.35	1887.07	5809.18	2676.2
	DQA1*02:01/DQB1*03:03	1353.97	586.31	884.5	3993.48	2377.55	2047.16	714.55	898.78
	DQA1*02:01/DQB1*05:02	5450.46	2704.78	6757.96	7184.29	12293.57	1790.02	4252.35	4105.86
	DRB1*07:01	499.74	142.44	1523.57	3327.24	1828.46	408.25	69.15	192.94
	DRB1*15:02	1718.84	643.06	4598.16	7086.16	3534.89	729.9	657.3	408.08
	DRB4*01:03	1226.69	356.5	1547.14	2871.31	1571.02	571.5	341.75	150.45
	DRB5*01:01	266.82	181.7	4061.68	2496.2	2671.39	122.72	50.14	147.14
Cell 2	DPA1*01:03/DPB1*04:01	5346.66	56.26	5351.99	12281.74	14299.56	402.82	1178.63	160.24
	DPA1*01:03/DPB1*04:02	5570.17	134.89	6399.86	13802.48	13670.5	616.85	1306.3	301.41
	DQA1*01:03/DQB1*03:01	660.9	688.82	505.07	2748.21	941.66	1259.07	422.21	849.99
	DQA1*01:03/DQB1*06:03	6139.27	2786.59	5494.27	14359.56	9980.83	5482.46	3386.45	2597.35
	DQA1*05:05/DQB1*03:01	791.41	1556.78	390.99	3335.9	797.41	1492.36	520.33	2714.43
	DQA1*05:05/DQB1*06:03	4086.54	2977.81	2609.04	8767.92	5526.59	3649.18	2602.95	3692.71
	DRB1*13:01	530.23	219.41	881.68	1098.35	376.13	290.34	77.93	80.6
	DRB1*14:06	916.37	449.54	1885.23	2547.11	759.16	673.89	261.91	170.57
	DRB3*01:01	2809.08	2342.87	9121.4	12353.04	6546.58	4696.51	2834.42	2978.81
